# ﻿Novel *in situ* observations of asexual reproduction in the carpet sea anemone, *Stichodactylamertensii* (Stichodactylidae, Actiniaria)

**DOI:** 10.3897/zookeys.1103.84415

**Published:** 2022-05-27

**Authors:** Morgan F. Bennett-Smith, Micaela S. Justo, Michael L. Berumen, Raquel Peixoto, Benjamin M. Titus

**Affiliations:** 1 King Abdullah University of Science and Technology, Red Sea Research Center, 4700 Thuwal, 23955 Saudi Arabia King Abdullah University of Science and Technology Thuwal Saudi Arabia; 2 Department of Biological Sciences, University of Alabama, Tuscaloosa, AL 35899, USA University of Alabama Tuscaloosa United States of America; 3 Dauphin Island Sea Lab, 101 Bienville Blvd, Dauphin Island, AL, 36528, USA Dauphin Island Sea Lab Dauphin Island United States of America

**Keywords:** Actinians, clonality, fragmentation, Indian Ocean, reproduction, sea anemones

## Abstract

Merten’s carpet sea anemone, *Stichodactylamertensii* Brandt, 1835, is the largest known sea anemone species in the world, regularly exceeding one meter in oral disc diameter. A tropical species from the Indo-Pacific, *S.mertensii* drapes prominently over coral reef substrates and is a common host to numerous species of clownfishes and other symbionts throughout its range, which extends from the Red Sea through the Central Pacific Ocean. Long thought to reproduce via sexual reproduction only, recent genetic evidence suggests it may rarely reproduce asexually as well, although this process had never been confirmed through direct observation and the mechanism was yet to be described. Here, we directly observed and documented *in situ* asexual fragmentation via budding, in real time, by a Red Sea *S.mertensii* in a turbid inshore reef environment. While asexual reproduction is not unusual in sea anemones as a group, it is typically expected to be uncommon for large-bodied species. Herein, we describe *S.mertensii* fragmentation, provide high resolution images of the event from the Saudi Arabian coastline at multiple time points, and confirm asexual reproduction for this species.

## ﻿Introduction

Asexual reproduction is common in sea anemones (Anthozoa, Actiniaria), which have evolved a variety of different asexual modes including pedal laceration, binary fission, longitudinal fission, and budding (reviewed by Shick 1991). Asexual reproduction can lead to small clusters of two or three anemones or to expansive clonal aggregations of hundreds of individuals. Clonality can thus make important contributions to sea anemone population dynamics, especially for tropical species that serve as symbiotic hosts to a diverse suite of fishes and other invertebrates.

The Red Sea contains thousands of kilometers of fringing coral reef systems inhabited by tropical sea anemones, the largest of which serve as symbiotic hosts to clownfish. Yet only recently has there been clarity on the diversity of host anemone species that inhabit this region (Bennett-Smith et al. 2021). The largest species found in the Red Sea, *Stichodactylamertensii* Brandt, 1835, is the largest known anemone species in the world, but has historically not been known to this region until only recently (Bennett-Smith et al. 2021). Although it is also possible that a range expansion has occurred, recent widespread documentation on surveys along the entire eastern coastline of the Red Sea indicates that *S.mertensii* is native to the region but remained unrecorded as a result of misidentifications in the literature (Bennett-Smith et al. 2021). In any case, despite *S.mertensii*’s widespread occurrence in the Red Sea, there have been few studies concerning its ecology or life history.

*Stichodactylamertensii* is one of ten described clownfish-hosting anemone species found on Indo-Pacific coral reefs (reviewed by Titus et al. 2019). Only two, *Entacmaeaquadricolor* (Leuckart in Rüppell & Leuckart, 1828) and *Heteractismagnifica* (Quoy & Gaimard, 1833) are known to reproduce clonally – a process well known to those in the aquarium trade who regularly propagate these species through binary fission by cutting the oral disc in half, resulting in two individuals. In the wild, *E.quadricolor* and *H.magnifica* regularly form clonal aggregations throughout their range via binary fission (Dunn 1981).

*Stichodactylamertensii* was thought to reproduce sexually, not asexually, following the generalization that it had only ever been found solitarily and that smaller, facultatively clonal animals are more likely to reproduce asexually compared to their larger counterparts (reviewed by Titus et al. 2017). Recent work in the Red Sea provided an indication of low levels of potential clonality in *S.mertensii* populations through genetic sampling. Out of 122 individuals sampled by Emms et al. (2020), two were determined to be potential clones and both were found in waters surrounding or adjacent to the Arabian Peninsula (Saudi Arabia & Djibouti). However, direct confirmation and mechanisms for asexual reproduction had not been documented until now. Here, for the first time, we observed fragmentation via budding from the column in real time in a Red Sea *S.mertensii*. We photographed the specimen at several time points to track its asexual reproduction *in situ*. This evidence offers insight into the reproductive mechanisms of clonality in this species and expands our general knowledge of reproductive modes for the clownfish-hosting sea anemones.

## ﻿Materials and methods

We conducted initial underwater surveys on SCUBA, near the campus of the King Abdullah University of Science and Technology (KAUST), in December 2021. During these surveys, we encountered several host anemone species, including *E.quadricolor* and *S.mertensii*.

To identify the host anemones located, we noted external morphological characteristics and used the dichotomous keys by Dunn (1981) and Fautin and Allen (1992). Morphological characteristics that were used to identify host anemones in the field included: the size and shape of the oral disc (flat, undulating, balled around the tentacles); the size, shape, color, and prevalence of verrucae (warty projections on the column) towards the pedal disc; the size, shape, density and uniformity of tentacles throughout the oral disc; the color pattern on the margins of the oral disc; the substratum on which the pedal disc was anchored (sand, rockwork, or rubble); and the coloration and appearance of the mouth. In the case of *S.mertensii*, this species has a large, flat oral disc, rounded, bulbous tentacle tips, longer tentacles around the mouth than at the periphery of the disc, and conspicuous verrucae along the column, extending to the pedal disc (Dunn 1981; Bennett-Smith et al. 2021).

**Figure 1.**
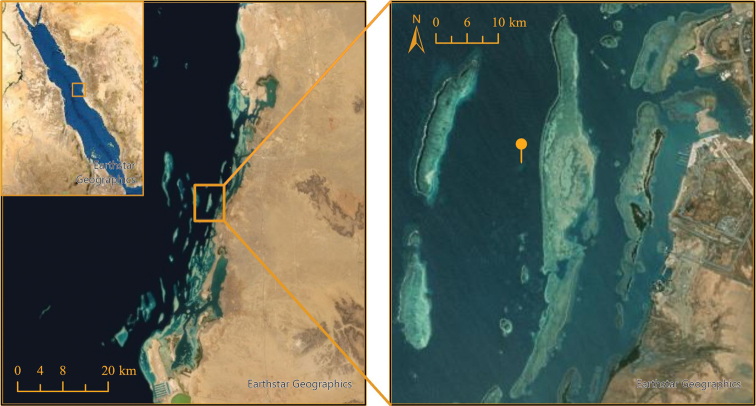
Location of observed *Stichodactylamertensii* on an inshore Red Sea reef near the King Abdullah University of Science and Technology; Thuwal, Saudi Arabia.

One anemone identified as *S.mertensii* was observed in the process of asexually fragmenting via column budding. This individual was subsequently GPS-marked, located at the following coordinates: 22°16'41.32"N, 39°3'54.23"E (Fig. 1). The anemone was identified at a depth of 11 meters. The anemone was photographed *in situ* with a Canon R5 camera inside a Nauticam underwater housing, with two Sea and Sea underwater strobes.

After our initial observations, we returned to the same location again in February 2022. We followed the same procedure and again photographed and measured the individual on SCUBA, using the same equipment.

## ﻿Results

### ﻿Description

**Initial observation**

**Figure 2.**
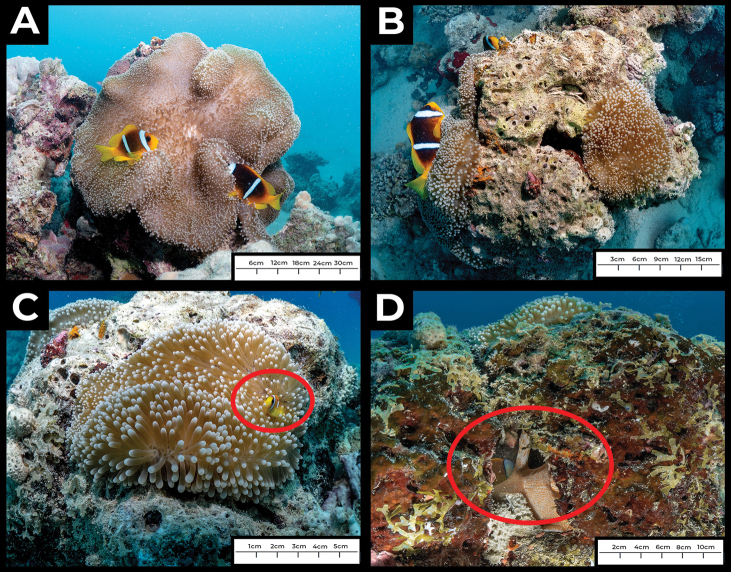
*In situ* images of asexual reproduction of *Stichodactylamertensii* on an inshore reef near Thuwal, Saudi Arabia **A** the parent *S.mertensii* individual, with two *Amphiprionbicinctus* symbionts **B** top view of the parent individual (left, with anemonefish) and newly forming anemone bud (right) **C** anemonefish recruit (circled) in newly forming anemone bud **D** cross section of the reef rockwork, showing the column of the anemone from where the new fragmentation branches.

**Figure 3.**
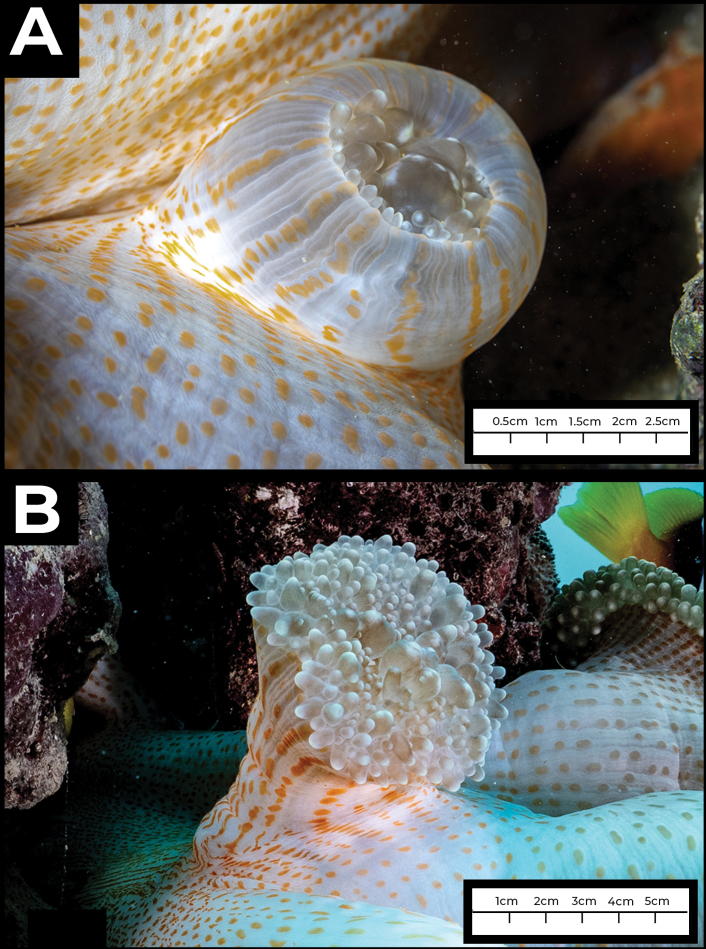
*In situ* macro images of new asexual bud on *Stichodactylamertensii* near Thuwal, Saudi Arabi at two time points **A** initial observation, December 2021; bud oral disc diameter ~2 cm **B** second observation, February 2022; bud oral disc diameter ~5 cm.

The individual that was observed had two separate budding locations, both on the column of the animal (Figs 2, 3). When first observed (December 8, 2021), one fragmentation bud was already recognizable as a separate individual, around 6 cm in length, extending outwards, with tentacles fully developed, even though it was still attached to the column. The other bud was small, less than 2 cm in oral disc diameter and newly formed, with tentacles not extended (Fig. 3A).

#### Second observation

The second observation was made on February 11, 2022 (33 days after the first observation). The larger of the two fragmentation buds had grown to an oral disc diameter size of ~12 cm, showing an increase of about 6 cm in oral disc diameter (Fig. 2B, C). The smaller bud had grown from an initial disc diameter size of less than 2 cm to around 5 cm, an increase of 3 cm (Fig. 3B).

Notably, the larger bud appeared to be much closer to separating from the parent entirely, and was only connected to the column by a thin stalk (Fig. 2D).

## ﻿Conclusions

These observations are the first of *in situ* asexual reproduction of *Stichodactylamertensii* (and the first of any carpet anemone species in the Red Sea), yielding insight into the mechanisms by which these species reproduce clonally. Interestingly, *Stichodactylamertensii* was not previously known to form clonal aggregations, and a recent survey effort covering several thousand km of Red Sea reefs did not reveal a single aggregation of any carpet anemone species (Bennett-Smith et al. 2021). Similarly, other large clownfish-hosting species from the genus *Stichodactyla*, like *Stichodactylahaddoni* (Saville-Kent, 1893) and *Stichodactylagigantea* (Forsskål, 1775), do not form aggregations of individuals and are not thought to reproduce asexually. However, Titus et al. (2019) found *H.magnifica*, a species well known to reproduce asexually, to be well nested within a broader clade containing the members of the genus *Stichodactyla*. Additionally, *S.helianthus*, a smaller carpet anemone species found on coral reefs in the Tropical Western Atlantic, is a clonal species as well. Thus, it is possible that this reproductive mode has been overlooked in the Indo-Pacific members of the genus *Stichodactyla*. Our observations in the Red Sea confirm *S.mertensii* as the third species of clownfish-hosting sea anemone known to reproduce asexually, along with *E.quadricolor* and *H.magnifica*. The asexual reproductive strategies of other host anemones from the Red Sea and elsewhere in the Indo-Pacific, including *Stichodactylahaddoni*, *S.gigantea*, *Heteractisaurora* (Quoy & Gaimard, 1833), *Heteractiscrispa* (Hemprich & Ehrenberg in Ehrenberg, 1834), *Heteractismalu* (Haddon & Shackleton, 1893) and *Macrodactyladoreensis* (Quoy, Gaimard, 1833), also remain unclear. Increased observational effort and further molecular work on this group may clarify these questions, which have downstream implications for a range of host anemone-associated taxa.
